# Factors associated with utilization of long acting and permanent contraceptive methods among married women of reproductive age in Mekelle town, Tigray region, north Ethiopia

**DOI:** 10.1186/1471-2393-12-6

**Published:** 2012-01-26

**Authors:** Mussie Alemayehu, Tefera Belachew, Tizta Tilahun

**Affiliations:** 1 Department of Public Health, Mekelle University, Mekelle, Ethiopia; 2 Department of Population and Family Health, Jimma University, Jimma, Ethiopia

## Abstract

**Background:**

Ethiopia is the second most populous country in Sub-Saharan Africa. Total Fertility Rate of Ethiopia is 5.4 children per women, population growth rate is estimated to be 2.7% per year and contraceptive prevalence rate is only 15% while the unmet need for family planning is 34%. Overall awareness of Family Planning methods is high, at 87%. The prevalence of long acting and permanent contraceptive methods (LAPMs) in Tigray region was very low which accounts for 0.1% for implants and no users for intra-uterine contraceptive device (IUCD) and female sterilization. Moreover almost all modern contraceptive use in Ethiopia is dependent on short acting contraceptive methods. The objective of this study was to assess factors associated with utilization of long acting and permanent contraceptive methods (LAPM) among married women of reproductive age group in Mekelle town.

**Methods:**

A cross sectional community based survey was conducted from March 9-20, 2011. Multistage sample technique was used to select the participants for the quantitative methods whereas purposive sampling was used for the qualitative part of the study. Binary descriptive statistics and multiple variable regressions were done.

**Results:**

The study consisted of quantitative and qualitative data. From the quantitative part of the study the response rate of the study was 95.6%. Of the qualitative part two FGDs were conducted for each married women and married men. 64% of the married women heard about LAPMs. More than half (53.6%) of the married women had negative attitude towards practicing of LAPMs. The overall prevalence of LAPMs use was 12.3% however; there were no users for female or male sterilization. The main reason cited by the majority of the married women for not using LAPMs was using another method of contraception 360 (93.3%). Mothers who had high knowledge were 8 times more likely to use LAPMs as compared with those who had low knowledge (AOR = 7.9, 95% CI of (3.1, 18.3). Mothers who had two or more pregnancies were 3 times more likely to use LAPM as compared with those who had one pregnancy (AOR = 2.7, 95% CI of (1.4, 5.1).

**Conclusion:**

A significant amount of the participants had low knowledge on permanent contraceptive particularly vasectomy. More than half (53.6%) of married women had negative attitude towards practicing of LAMPs. Few of married women use female sterilization and none use of female sterilization and or vasectomy. Positive knowledge of LAMPs, women who had two and above pregnancies and women who do not want to have additional child were significantly associated. Information education communication should focus on alleviating factors hinder from practicing of LAPMs.

## Background

An estimated 358 000 maternal deaths occurred worldwide in 2008, a 34% decline from the levels of 1990. Despite this decline, developing countries continued to account for 99% (355 000) of the deaths. Sub-Saharan Africa and South Asia accounted for 87% (313,000) of global maternal deaths [1]. Fortunately, the vast majority of maternal and newborn deaths can be prevented with proven interventions to ensure that every pregnancy is wanted using modern contraceptive and every birth is safe [2].

Women and couples who want safe and effective protection against pregnancy would benefit from access to more contraceptive choices, including long acting and permanent contraceptive methods (LAPMs). Despite of these advantages, LAPMs are given in few areas and sometimes are missing component of many national reproductive health and family planning programs. More than 350 million couples worldwide have limited or no access to effective and affordable FP, especially to LAPMs [3]. Thirteen percent of the world's married women use the Intrauterine Contraceptive Device (IUCD) as their method of contraception [4].

In developing countries, 20 to 30% of women who use oral contraceptives or injectables stop within two years of starting because of side effects or other health concerns. Many of these women could benefit from switching to LAPMs [5]. In Sub-Saharan Africa a quarter of women and couples have unmet needs for contraception [6-8], 14% used modern contraceptives and most births in the region are still spaced closer than two years [8,9].

Ethiopia is the second most populous nation in Africa. Its population has increased nearly seven times from 11.8 million at the beginning of the 20th century to about 80 million today [10]. The total fertility rate of Ethiopia is 5.4 children per women, population growth rate is estimated at 2.7% per year, contraceptive prevalence rate (CPR) is only 15% and an unmet need for family planning is 34 percent. Implants and female sterilization are the least used methods of modern contraceptive each accounting only for 0.2% [11]. Addressing the unmet need of family planning in Ethiopia is expected to avert 12,800 maternal deaths and more than 1.1 million child deaths by the target date of 2015 [12]. The prevalence of LAPMs use in Tigray region is rather very as low as 0.1% for implants and no users for IUCD and female sterilization [10]. The overall prevalence of any contraceptive use in Mekelle town is only 37% [13].

A number of factors could contribute to the lack of availability and access to LAPMs. Evidences show from other countries and within Ethiopia showed that many factors including fertility related reason, opposition to use, lack of knowledge, method related reason could act as barriers to LAMPs use [10]. Higher cost to individuals or the ministry of health (MOH); lack of trained providers and wide availability of short acting methods in the rural areas where most people live and distance to clinics and medical barriers inhibit access [9]. Health personnel may not provide LAPMs to clients because of unnecessary or outdated restrictions, such as age or the number of children a woman has [3]. Myths and misconceptions are also widespread for these methods [14].

There is no study that documented factors associated with very low use of LAMPs in Tigray region. This study was to assess factors associated with utilization of long acting (Implant and IUCD) and permanent (Vasectomy and Female sterilization) contraceptive methods among married women of reproductive age (15-49 years) in Mekelle town, Tigray region, North Ethiopia.

## Methods

### Study area and setting

A community based cross-sectional study was conducted from March 9-20, 2011. The study was done in Mekelle town, Tigray region, in the northern part of Ethiopia. The total population of the area is 233,000, with 112,250 males and 119,800 females [13]. Tigre is the dominant ethnic group in Mekelle. In Mekelle town the administration is divided in to districts (locally known as Ketna).

### Sampling

A sample of 460 married women of reproductive age was participated in the study. The sample size was determined using a formula for estimation of single population proportion with the assumption of 95% confidence level, margin of error of 5% and expected prevalence of modern contraceptive use Tigray region of (0.16) [10]. To compensate the non-response rate, 10% of the determined sample was added up on.

A multi-stage sampling technique was used to select the study participant by considering a design effect of two. The town is divided into 72 districts comprises of 54,073 households. Out of this 17 districts (consists 2,162 households) were selected using simple random sampling then proportion to size to districts was employed to share the sample size.

Then picking a house on random for the initial household from each sub-districts, the final households with married women were selected on systematic random sampling from the existing sampling frame of households. For selecting the study participants' different sampling intervals were used for each sub-district. Finally, eligible married women (15-49 years) were interviewed from each selected households. When two or more married women were encountered in one household, only one woman used to be considered in the study on random to avoid intra-class correlation.

Two sessions of married women and 2 married men focus group discussions (FGD) were organized based on information saturation. Each FGD consisted of 8 participants composed of married women of reproductive age group and married men. Selection was performed by purposive sampling technique taking into consideration sex and educational status for convenient individuals during FGD. FGD consist of similar ideas with questionnaire but also the guide-line help more to probe ideas which is difficult to collect using only quantitative methods i.e beliefs and attitude questions.

### Method of data collection

The quantitative data were collected using structured interviewer administered questionnaires and focus groups discussions were organized for the qualitative part. For quantitative data questionnaires were constitute information on socio-demographic and economic variables, reproductive history, knowledge, attitude and practice of LAPMs and family planning questions. The questionnaires were adapted from different studies considering the local situation of the study area [(Getachew M: Assessment of the prevalence and factors affecting use of permanent and long acting contraceptive methods in Jinka town, south Omo Zone, SNNPR, Ethiopia, submitted to Addis Ababa University), 15]. Questionnaires were prepared first in English then translated to local language (Tigrigna) for data collection by language expert. To check whether the translation was consistent with the English version the questionnaire were back translated to English by another language expert.

Before the actual data collection, the questionnaire was pre-tested on 5% (35 women) in Wukro town which is 45 km north from Mekelle. Based on the pre-test, the time needed for the complete interview and the number of data collectors in need was estimated. The principal investigator trained fifteen clinical nurse students as data collectors and five urban health extension workers as supervisors for two consecutive days on objective, data collection tools and interview techniques. The interview was conducted in a place where the woman feels free to express her feelings and ideas. Moreover, in occasions where the sampled women were not accessed for absence, up to three attempts were being endeavored for interviewing to lessen the non-response rate. The questionnaires were checked by the supervisors on daily basis for completeness.

Focus group guide line was used to explore ideas of male partners and married women on using LAPMs and for triangulation with the quantitative study. During the FGDs, participants were informed about the purpose and process of the FGD to obtain informed consent of each participant. Two persons were assigned for note taking and tape recording while the principal investigator facilitated the discussion. Two FGD were conducted separately each for males and females, consisting of eight participants each. Each FGD took an average of 1 to 1 and a half hour. Some of the issues raised on FGDs were: the attitudes towards practicing LAPMs, reasons for not practicing LAMPs, when the women prefer to start LAMPs and its reasons. Finally, the recorded discussions and notes were transcribed into English manually.

### Measurement

Married women's knowledge was measured by the total number of correct answers to 10 items on knowledge with a minimum score of 0 and maximum of 10. To measure the knowledge it was categorized based on the percent of knowledge of the distinct characteristics of LAMPs as: "high" - those who knew 80% and above, "moderate" those who know 60 - 79% and "low" those who knew less than 60%.

Items on attitude of married women about the use of LAPM were grouped in to three as follows: "strongly agrees/agree" were labeled as "agree" and "strongly disagree/disagree" as "disagree", while "not sure" was categorized as it is. To measure the attitude of the married women two categories were assigned: Positive Attitude - those who scored above the mean on attitude items and "Negative Attitude" - those who scored the mean or below mean to attitude items.

For analyzing the attitude, married women to the use of LAPM were grouped into three "strongly agrees" and "agree" were grouped together as "agree", "strongly disagree" and "disagree" were grouped together as "disagree" while not sure is categorized as it is. To measure the attitude of the married women, two categories were assigned: Positive Attitude - those who scores above mean to the correct answers from attitude measuring LAPMs questions. Negative Attitude - those who score mean and below mean to the correct answers from attitude measuring LAPMs questions. Finally, married women's use or not use of long acting and permanent contraceptive methods among study units was set as binary outcome variable.

### Data analysis

The quantitative data were entered, cleaned and analyzed by using Statistical package for social sciences (SPSS) version 16.0 (SPSS Illinois, Chicago). First descriptive analyses were carried out for each of the variables. Second, bivariate analyses were done for the independent variables with the outcome variable to select candidate variables for the multivariable analyses.

Finally, Variables which showed significant association with the dependent variable on the bivariate analysis were entered to multivariate logistic regression model to identify their independent effects. Data from the focus group discussion were translated and transcribed to English and categorized accordingly to main thematic areas manually. The findings were presented in narratives in triangulation with the quantitative results using the well said verbatim as illustrations.

### Ethical considerations

Ethical clearance was obtained from Jimma University; college of Public Health and Medical Sciences Ethical Committee. Informed consent was obtained from each study participant.

## Results

The total response rate of the survey was 95.6%, out of 460 married women. The mean age of the married women was 29.42 (SD = 6.94) years. The majorities were Orthodox Christians (93.9%), had attended formal education (84.5%) and most (53.4%) were house wives. The mean monthly income of the family was $69 (SD = 14). Out of the total married women 392 (89.1%) and 336 (76.4%) had radio and television respectively (Table 1).

**Table 1:** Socio-Demographic Characteristics of Married Women, Mekelle town, 2011

Variables	Number	Percent
**Age of married women (n = 440)**		

15-24	115	26.1
25-34	208	47.2
35-44	98	22.2
> = 45	19	4.3

**Religion of married women (n = 440)**		

Orthodox	413	93.9
Islam	23	5.2
Others (Catholic and Protestant)	4	0.9

**Education status (n = 440)**		

Not able to read and write	68	15.5
Able to read and write	32	7.3
1-8 grade	166	37.7
9-12 grade	124	28.2
College or University level	50	11.4

**Occupational status (n = 440)**		

Student	39	8.9
Merchant	105	23.9
Governmental or nongovernmental worker	34	7.7
Daily labor	27	6.1

**Monthly income (n = 440)**		

< 31.24 $	126	28.6
31.25-62.49$	183	41.4
≥ 62.50 $	132	30

**Have radio & or TV (n = 440)**		

Have radio	392	89.1
Have TV	336	76.4

**Practice of LAPM (n = 440)**	54	12.3

**Type of LAPM (n = 54)**		
Implant	47	87
IUCD	7	13

1$ = 17 Ethiopian Birr

The overall prevalence use of long acting and permanent contraceptive methods use was 12.3%. The majority of women used implants (87%) followed by IUCD (13%). There were no married women who underwent female sterilization. The results of FGD also support this. A focus group participant said *"...I heard about male contraceptive method just now. Only females are using all methods like Implant, IUCD and female sterilization still now. Even, there are males who do not want their wife to use any type of contraceptive methods..." *[32 years old, male, orthodox]. The prevalence of implants and IUCD users was 10.6 and 1.5% respectively. The average year of practicing LAPM was 2.39 (SD = 1.2), of which, 2.34 (SD = 1.2) for implants and 2.71 (SD = 1.6) for IUCD. The majority (83.3%) of the married women gets the service from public institution, GO 6 (11.1%) and private organization 3 (5.6%). Eighty three point three percent of the married women got the contraceptive for free, while 16.7% paid to get it and all the married women told that the fee was affordable.

The main reason cited by the married women for not using LAPM was the use of another method of contraceptive 360 (93.3%), developing side effect 15(3.9%) and not allowed by 6 (1.6%) husband and medical problem and the non availability of service) 5(1.3%). The mean age of women at marriage and first delivery was 18.92 (SD = 2.7) and 20.8 (SD = 2.8) years, respectively. The average number of pregnancies was 2.16 (SD = 0.9), out of which, (56.6%) experienced one pregnancy. Sixty-three (14.3%) of the married women had abortion, of which 30.2% experienced more than one induced abortion in their life time. The average number of children in the household was 1.28 (SD = 0.5) (Table 2).

**Table 2:** Reproductive History of Married Women, Mekelle town, 2011

Variables	Number	Percent
**Age at marriage (n = 440)**		

< 18	238	54.1
≥ 18	202	45.9

**Age at delivery (n = 421)**		

< 18	71	16.9
≥ 18	350	83.1

**Number of pregnancy (n = 440)**		

One	249	56.6
Two and above	191	43.4

**Number of abortion (n = 63)**		

One	44	69.8
Two and above	19	30.2

**Responsible for deciding to have children (n = 440)**		

Wife	73	16.6
Husband	14	3.2
Joint discussion	353	80.2

Regarding married women general awareness about LAMPs, 63.9% had heard about LAPMs in general, out of this, 80.7%, 55.3% and 39.8% had heard about implants, IUCD and female sterilization, respectively. Only 15.6% of the married women heard about vasectomy and 23.8% named more than two contraceptive. Moreover, 124 (44.1%) had awareness about more than one advantage of LAPMs. A focus group discussant said *"... Mostly, community members had awareness about Implant. However, I don't think that they have better awareness about loop and female sterilization. Moreover, this is my first time to hear about male sterilization ..." *[32 years old, female, Orthodox]. Among the married women 77.5% and 125(44.4%) had *awareness *on the advantage of LAPM for prevention of unwanted pregnancy and helps to have planned family. Likewise, finding from a focus group participant stated *"... LAPM has a lot of advantage such as reduce infant and maternal mortality and increase the economy capacity of the household, community and the country as a whole..." *[41 years old, male, Orthodox]. The sources of information for LAMPs among the women were public institution 154 (54.8%), mass media 59 (21%), family 50 (17%) and combination of these sources 18 (6.4%).

One hundred eleven (37.8%) of the women were aware of that IUCD can prevent pregnancies for 10 years and 42.5% were not sure of if IUCD is good for female at risk of acquiring sexual transmitted infection. In this study 48% and 62.2% of the women aware of that IUCD has no influence on sexual intercourse and it results in immediate pregnancies after removal, respectively. The majority (69.7%) of the married women aware of that implants result in immediate pregnancy after removal. 126 (45%) of the married women were in the category of low knowledge, followed by high knowledge 137 (31.1%) towards LAPM where as the remaining (23.7%) had moderate knowledge. Ninety seven (33%) of the married women knew that male sterilization has no influence on sexual intercourse, while less than three fourth (23.8%) of the married women were aware that pregnancy is not possible after tubal ligation is done for female sterilization (Table 3).

**Table 3:** Knowledge of Married Women about Long Acting and Permanent Contraceptive Methods of Mekelle town, 2011 (N = 281)

S.No	Knowledge statements	Knowledge of married women on LAPM		
		**True**	**False**	**Not sure**
		%	%	%
	
**1.**	IUCD can prevent pregnancies for more than 10 years	37.8	10.9	51.4
**2.**	IUCD is not appropriate for female at high risk of getting STIs	29.9	27.6	42.5
**3.**	IUCD has no interference with sexual intercourse or desire	48	10.9	41.2
**4.**	IUCD is immediately reversible(become pregnant quickly when removed)	62.2	14.6	23.1
**5.**	IUCD cannot cause cancer	57.5	30.6	11.9
**6.**	Implant can prevent pregnancies for 5 years	89.5	4.4	6.1
**7.**	Implants require minor surgical procedure during insertion and removal	87	6.1	6.8
**8.**	Implantsis immediately reversible(becomes pregnant quickly when removed)	69.7	13.3	17
**9.**	Vasectomy has no interference with sexual intercourse	33	8.2	58.8
**10.**	After female sterilization pregnancy is not possible	23.8	45.6	30.6

With regard to attitudes about LAMPs, 15.5% and 26.8% married women agreed that implant can result in irregular bleeding and cause severe pain during insertion and removal *respectively*. Above one fourth (29.7%) of the married women agreed that insertion of IUCD can result in shame while it inserted to cervix by health professional. Nineteen point six percent agreed that IUCD prevents from doing normal activities and 34.4% agreed that undergoing an operation for female sterilization was dangerous. Asked on their attitudes about the side effects of LAMPs, they agreed that irregular bleeding due to the use of implant is severe (28.9%), insertion and removal of implant is highly pain full (33.1%), losing privacy during IUCD insertion is shameful (65.3%) and undergoing operation for female sterilization is unacceptable (39.1%) (Table 4).

**Table 4:** Attitude about and Their Side Effects of Long Acting and Permanent Contraceptive Methods Mekelle town, 2011 (N = 440)

		Disagree		Not sure		Agree	
S.No		No	%	**N**o	%	**N**o	%
	**Attitude about LAMPs**						
**1**	Using implant cause irregular bleeding	118	26.8	254	57.7	68	15.5
**2**	The insertion and removal implant is highly pain full	76	17.3	246	55.9	118	26.8
**3**	Insertion of Intra uterine contraceptive device cause to lose privacy	229	52.1	80	18.2	133	29.7
**4**	Using Intra uterine contraceptive device restrict normal activities	145	32.9	209	47.5	86	19.6
**5**	Operation for female sterilization is dangerous	68	15.4	231	50.2	151	34.4

	**Attitudes statements about side effects of LAMPs**						

**6**	For me irregular bleeding due to using implant is severe	95	21.6	218	49.5	127	28.9
**7**	For me insertion and removal of implant is highly pain full	91	20.7	203	46.1	146	33.1
**8**	For me loosing privacy during Intra uterine contraceptive device insertion is shame full	89	20.3	64	14.5	187	65.3
**9**	For me by using Intra uterine contraceptive device restricted from different work activity highly un acceptable	60	13.7	131	29.8	249	56.6
**10**	For me operation for female sterilization is unacceptable	84	19.1	184	41.8	172	39.1

Concerning the level of attitudes, more than half (53.6%) of the married women had negative attitude towards practicing of LAPM. *This can be illustrated by what *a focus group participant said as follows *"...I think it is best to practice long acting contraceptive however I don't encourage use of permanent contraceptive., If someone has female sterilization, she cannot give birth and her husband may divorce her and she may encounter a problem to raise her child, so why do we encourage the utilization of it. In my opinion, nobody should use it..." *[31 year's old, male, Orthodox]

The results of multivariable logistic regression analysis showed that women who had moderate knowledge were 6 times more likely to use LAPM as compared with those who had low knowledge (AOR = 5.9, 95% CI: 2.3, 14.9). Mothers who had high knowledge were 8 times more likely to use LAPM as compared with those who had low knowledge (AOR = 7.8, 95% CI: 3.1, 18.3). Mothers with two or more pregnancies were 3 times more likely to use LAPM as compared with those who had been pregnant only once (AOR = 2.7, 95%: 1.4, 5.1). As the delivery age of the mother increase by one year the use of long acting and permanent contraceptive also increased twice (AOR = 2.1, 95% CI: 1.8, 2.9)(Table 5).

**Table 5:** Predictors of Use Long Acting and Permanent Contraceptive Methods of Mekelle town, 2011.

Variables (n = 440)	Use of LAPM		Adjusted OR
	Yes	No	
	No(%)	No(%)	

Knowledge			

Low Knowledge	7(12.9)	192(87.1)	1
Moderate Knowledge	19(35.1)	85(64.9)	5.9(2.3,14.9)
High Knowledge	28(51.8)	109(48.2)	7.5(3.1,18.3)

Number of pregnancy			

One	22(40.7)	227(59.3)	1
Two and above	32(59.3)	159(41.1)	2.7(1.4,5.1)

Age category			1

15-24	7(12)	108(88)	2.3(0.4,46.6)
25-29	16(29.6)	110(70.4)	2.9(0.2,20.1)
30-34	10(18.6)	72(83.4)	1.4(0.5,44,8)
35-39	16(29.6)	44(70.4)	3.20(0.1,18.9)
≥ 40	5(7)	34(93)	1

Desire for more children			

Don't want	29(53.7)	143(46.3)	2.5(1.4, 5.1)
want	25(42.3)	243(57.7)	1

(N = 440)

## Discussion

The result showed that 64% of the married women heard about LAPM and 81%, 55% and 40% heard about implants, IUCD and female sterilization respectively which is higher as compared with finding three studies conducted in Ethiopia: the EDHS 2005 and a study conducted in Jinka and Butajira [11, Getachew M: Assessment of the prevalence and factors affecting use of permanent and long acting contraceptive methods in Jinka town, south Omo Zone, SNNPR, Ethiopia, submitted to Addis Ababa University, Temesgen A: Assessment of the prevalence and factors influencing the utilization of long acting and permanent contraceptive method in Butajira town, Gurage zone, SNNP, Ethiopia, submitted to Addis Ababa University]. This might be due to the difference in resident of the study participants, availability of urban health extension workers and continuous advertisement of these contraceptives through media (TV and radio) in the study area which is an urban setting.

In this study the overall prevalence of use LAPM was 12% however, which is higher than the prevalence reported from Jinka (7%), Butajira (5%) and the report of EDHS 2005 [Getachew M: Assessment of the prevalence and factors affecting use of permanent and long acting contraceptive methods in Jinka town, south Omo Zone, SNNPR, Ethiopia, submitted to Addis Ababa University, Temesgen A: Assessment of the prevalence and factors influencing the utilization of long acting and permanent contraceptive method in Butajira town, Gurage zone, SNNP, Ethiopia, submitted to Addis Ababa University,11]. Which might be due to difference in the study areas, access to information and the services as the three studies used different levels of coverage in terms of the study population?

Unlike the high prevalence of request for re-insertion of Norplant after five years of use, in other developing countries like Singapore (53%), our results show a very low rate (11%). In connection to this the degree of satisfaction was rated to be "very good" by 46% subject and "good" by 31%. The main reasons mentioned for switching to implant contraceptive among subjects were; convenience (64%), contraceptive failure (11%) and experienced side-effects with other contraceptive methods (36%) [16]. This might be due to the fact that in our study 27% of the married women believe that implants insertion and removal is very painful as well as difference in the socio economic status of the married women and nature of the study area. Conversely, the findings were higher compared with findings from EDHS 2005 and studies done in Jinka and Butajira [11, Getachew M: Assessment of the prevalence and factors affecting use of permanent and long acting contraceptive methods in Jinka town, south Omo Zone, SNNPR, Ethiopia, submitted to Addis Ababa University, Temesgen A: Assessment of the prevalence and factors influencing the utilization of long acting and permanent contraceptive method in Butajira town, Gurage zone, SNNP, Ethiopia, submitted to Addis Ababa University]. This might be due the married women in this study have more accessible to service use.

Only 1.5% of the married women used IUCD, which is lower as compared with a study done in Nigeria (7%) and Indonesia (52%) [17,18]. Which might be due to the fact that large number of the women had misconception about IUCD and its side effects such as interference with sexual intercourse, cancer, delays pregnancy, restriction from working normal activity and invasion of privacy during its insertion and removal. The main reason perceived for not practicing IUCD was the use of other methods of contraceptives, fear of side effects, cause cancer and husband's disapproval which is supported by reports from Elsalvadore and Turkey [15,19].

Similar to the findings of EDHS and a report from Arsi [11,20], there were no users of female sterilization in Mekelle town. However, this finding is not consistent with other studies which showed higher rates of sterilization in Jinka 36%), Butajira (10%), Caribbean (4%) and Uzbekistan (5%) [Getachew M: Assessment of the prevalence and factors affecting use of permanent and long acting contraceptive methods in Jinka town, south Omo Zone, SNNPR, Ethiopia, submitted to Addis Ababa University, Temesgen A: Assessment of the prevalence and factors influencing the utilization of long acting and permanent contraceptive method in Butajira town, Gurage zone, SNNP, Ethiopia, submitted to Addis Ababa University, 21, 22]. A study done in England showed that women prefer female sterilization because it is irreversible, does not involve hormonal treatment and they do not wish to have child for the future [23]. This might be due to the fact that large number (54%) married women who had negative attitudes towards it and their great interest to have children in the future (61%).

In this study women large proportion of women use contraception for child spacing (65%) than permanent limitation for number of children (17%) which the reverse of the what was reported from Nigeria [17] where, 30% of women contraception for child spacing versus 70% use it for permanent limitation for number of children. This might be related to the fact that large number (61%) of the married women having an interest a child for the future as well as majority of the married women don't have positive attitude towards permanent methods of the contraception in the study community. The major source to obtain contraceptives for the married women was a public health facility (83%). This finding is consistent with findings of EDHS 2005(80%), Butajira(80.8%) and Jinka (> 80%) [11, Temesgen A: Assessment of the prevalence and factors influencing the utilization of long acting and permanent contraceptive method in Butajira town, Gurage zone, SNNP, Ethiopia, submitted to Addis Ababa University, Getachew M: Assessment of the prevalence and factors affecting use of permanent and long acting contraceptive methods in Jinka town, south Omo Zone, SNNPR, Ethiopia, submitted to Addis Ababa University].

Most participants of the focused group discussions suggested that the optimum family size should be four which is parallel with other studies done in big regions of Ethiopia [24]. However, it is relatively inconsistent with finding of EDHS 2005 in which three out of five women preferred an ideal family size of four or more children [11].

Results of regression analysis showed that women who had high knowledge about LAPM were 8 times more likely to practice LAPM as compared with those had low knowledge which is consistent with a study done in Jinka and Butajira [Getachew M: Assessment of the prevalence and factors affecting use of permanent and long acting contraceptive methods in Jinka town, south Omo Zone, SNNPR, Ethiopia, submitted to Addis Ababa University, Temesgen A: Assessment of the prevalence and factors influencing the utilization of long acting and permanent contraceptive method in Butajira town, Gurage zone, SNNP, Ethiopia, submitted to Addis Ababa University].

Although statically not significant, married women with positive attitude towards LAMPs had the highest intention to use long acting contraceptives which is supported by another study that showed that positive attitude of women to contraceptive was an important factor for promoting use of long acting contraceptive [17]. The majority users of LAPM had joint discussion with their husband about number of children and were those who had radio and/or TV which is consistent with findings from Ethiopia and Indonesia [20,18].

Ethiopia had developed family planning including long acting and permanent contraceptive methods guideline with clear objectives concerning the indication of these contraceptive. For the implementation of these objective a few health professional were trained and providing varying level long acting contraceptive. The ultimate goal of this intervention is to increase community awareness, to have planned family and reduce children and maternal mortality. But still the coverage and practicing of LAMPs hang about at unsatisfactory level. Moreover, practicing of LAMPs has been affected by accessibility, availability and presence of trained health professional. Therefore, this may provoke policy makers and decision makers, to renew the intervention that is being given.

The study suffers from the usual limitation of a cross sectional study. The knowledge and attitude towards LAPMs among men and influence on their wives were not addressed through in this study. The study also did not discover any information from the service providing side of LAPMs. In addition, due to the nature of the sensitivity of some issues (like abortion) the information collected might not reflect the truth. However, study team had made an effort dig out possible information and to make the interview as private as possible to minimize such biases.

## Conclusion

A significant amount of the participants had low knowledge on permanent contraceptive particularly vasectomy. More than half of married women had negative attitude towards practicing of long acting and permanent contraceptive methods particularly for female sterilization and vasectomy. Few of married women use female sterilization and none use of female sterilization and or vasectomy. Positive knowledge of LAMPs, women who had two and above pregnancies and women who do not want to have additional child were significantly associated. The findings have implications for family planning programs to seriously examine ways to increase contraceptive use for those specifically on LAMPs. Continuous health education on LAPMs, increasing availability of LAPMs services in public and private institutions and information education communication should focus on addressing the needs of long acting and permanent contraceptive methods. It is therefore working in collaboration with non-governmental organizations and local community organizations are important. Furthermore further study should be conducted to produce better evidence focusing on the service providers, male partners, service delivering institutions and to identify factors influencing the utilization of LAPMs.

## Competing interests

The authors declare that they have no competing interests.

## Authors' contributions

MA, TB and TT designed the study, analyzed the data drafted the manuscript and critically reviewed the article.

All authors read and approved the final manuscript.

## Pre-publication history

The pre-publication history for this paper can be accessed here:

http://www.biomedcentral.com/1471-2393/12/6/prepub
